# Supplementary material for: The neural origin for asymmetric coding of surface color in the primate visual cortex
Source: Nat Commun. 2024 Jan 15;15:516. doi: 10.1038/s41467-024-44809-y (PMC10789876; doi:10.1038/s41467-024-44809-y)
Supplement: Supplementary file 1 — Supplementary Information [file 41467_2024_44809_MOESM1_ESM.pdf]

The neural origin for asymmetric coding of surface color in the primate visual cortex

Supplementary Information

Supplementary Note 1

Defining ROIs based on retinotopic probabilistic map Firstly, we aligned our data with another dataset shared by Janssens et al. (3) to shift the putative areal boundaries. Note that this dataset provides the probabilistic map of V1, V2, V3, and V4 based on the retinotopic maps from multiple monkeys. As shown in Supplementary Fig. 6, the ROIs defined in the main results showed a high probability of representing V1, V2, and V4. To avoid potential impacts of alignment accuracy (particularly around the boundaries) and individual differences, we selected voxels demonstrating at least 75% probability within the defined ROIs in the main text to create a new set of ROIs with higher precise representations of V1/V2/V4. Subsequently, we performed the same analyses in the main text, yielding similar findings to the main results (Figure 3). Please refer to Supplementary Fig. 7 for details.

Secondly, to shift the putative areal boundaries, instead of using the V1/V2/V4 masks from the D99 atlas, we selected voxels demonstrating at least 75% probability to create 75% probability V1/V2/V4 masks based on the probabilistic map. Note that the new set of masks may exhibit V1/V2/V4 more precisely. Next, we defined ROIs within these masks following the same methods as in the main text. Again, consistent results were found with those shown in the main Results (Supplementary Fig. 8).

Supplementary Note 2

Luminance-matching experiment We defined another set of stimuli based on the neural response from the M pathway (i.e., L4Cα & L4B in V1) in the luminance-matching experiment to roughly equate the subjective luminance between the wavelength-varying stimuli. In the luminance-matching experiment, we used a fast square-wave drifting grating (SF = 2 Hz, temporal frequency = 16.67 Hz) composed of background gray (10.6 cd/m2) and red/green/blue/yellow with multiple

levels of luminance. Based on the responses from the M pathway, for each color, we chose the luminance value that evoked the minimal response from L4Cα & L4B (dashed line in Supplementary Fig. 10) as the estimated "subjectively equal luminance". This procedure mimics the flicker photometry method in humans [1,2]. Then, we measured the V1 laminar response activated by uniform squares with color luminance defined as "subjectively equal luminance". In this way, we minimized the contamination of the response from luminance-selective cells in the M pathway. We show the laminar results of the luminance matched by the M pathway together with the laminar results of physically equal luminance in Figure Supplementary Fig. 11 to make a direct comparison.

Supplementary Tables

Supplementary Table 1 Parameters of stimuli in multiple color spaces in the electrophysiology and fMRI experiments

	Electrophysiology				fMRI			
	Hue(°)	RGB	CIE1931 (x, y, L)	Cone Contrast (L,M,S)	Hue(°)	RGB	CIE1931 (x, y, L)	Cone Contrast (L,M,S)
**Red**	0	(160.7, 0, 0)	(0.61, 0.35, 10.99)	(0.32, -0.31, -0.91)	0	(176, 0, 0,)	(0.65, 0.34, 5.20)	(0.27, -0.47, -0.95)
**Green**	120	(0, 45, 0)	(0.29, 0.59, 10.44)	(-0.05, 0.35,-0.84)	120	(0, 130, 0)	(0.38, 0.57, 5.17)	(-0.03, 0.20, -0.89)
**Blue**	240	(0, 0, 255)	(0.15, 0.07, 10.02)	(-0.24, -1.80, 6.90)	240	(0, 0, 255)	(0.14, 0.09, 5.15)	(-0.23, -1.20, 9.83)
**Yellow**	60	(36.5, 36.5,0)	(0.39, 0.52, 10.60)	(0.03, 0.22, -0.86)	60	(117.3, 117.3, 0)	(0.45, 0.49, 5.22)	(0.03, 0.07, -0.84)
**Black**	-	(0, 0 ,0)	(0.24, 0.19, 0.08)	(-0.99, -1.00, -0.98)	-	(0, 0 ,0)	(0.38, 0.33, 0)	(-1, -1, -1)
**White**	-	(61.2, 61.2, 61.2)	(0.28, 0.31, 20.40)	(0.93, 0.92, 0.96)	-	(137.7, 137.7, 137.7)	(0.35, 0.34, 10.1)	(0.96, 0.79, 1.36)
**Gray**	-	(30.6, 30.6, 30.6)	(0.28, 0.31, 10.55)	(0, 0, 0)	-	(117.3, 117.3, 117.3)	(0.34, 0.37, 5.22)	(0, 0, 0)

Supplementary Table 2 The correlation strength between LGN and V4 when controlling for V1 and/or V2.

	Controlled variables	r(p)		
		**Red–Green bias**	**Blue–Yellow bias**	**Black–White bias**
**LGN-V4**	-	0.330 (*p*<0.001)***	0.439 (*p*<0.001)***	0.509 (*p*<0.001)***
**LGN-V4**	V1	0.260 (*p*=0.010)*	0.355 (*p*<0.001)***	0.385 (*p*<0.001)***
**LGN-V4**	V2	0.175 (*p*=0.087)	0.326 (*p*=0.001)**	0.393 (*p*<0.001)***
**LGN-V4**	V1&V2	0.168 (*p*=0.101)	0.290 (*p*=0.004)	0.352 (*p*<0.001)***

*$p < 0.05$, **$p < 0.01$, ***$p < 0.001$, two-sided Spearman correlations.

Supplementary Figures

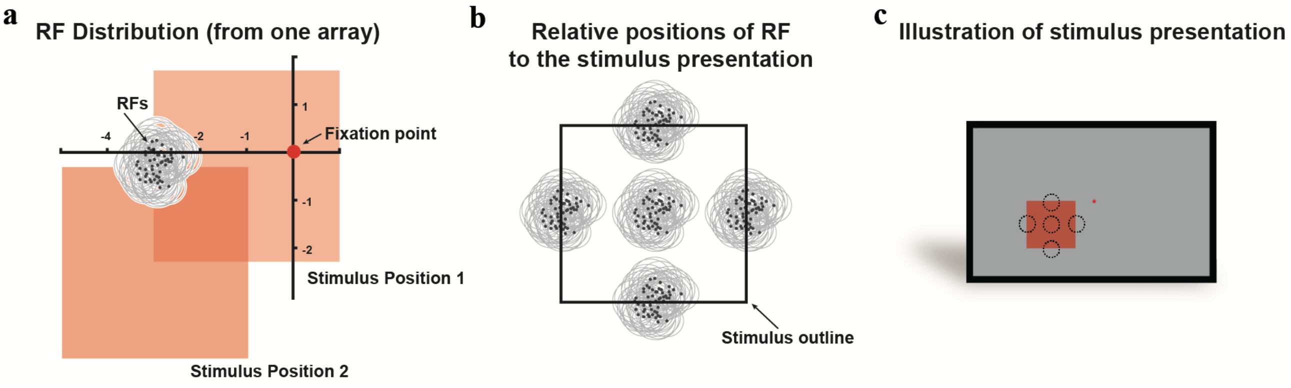

Supplementary Fig. 1 Illustration of stimulus presentation and RF distribution.

a. RF centers and sizes from one array of monkey DQ. Red squares represent two of five possible stimulus positions (left, right, top, down edge of a square in the RFs, surface center in the RFs). **b.** 5 relative positions of RF to the stimulus outline. **c.** Simplified illustration of stimulus presentation and RF locations relative to the stimulus.

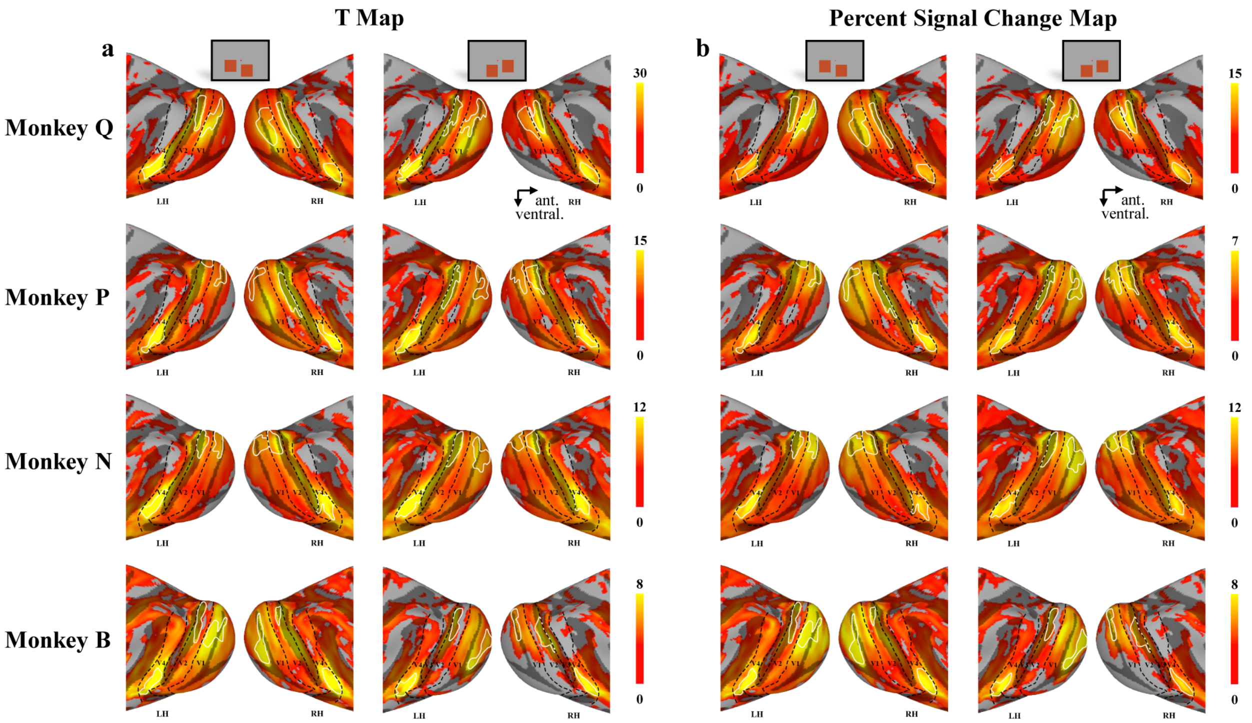

Supplementary Fig. 2. **Unthresholded t-map (a) and percental signal map (b) for each animal shown on lateral views of the inflated cortex of the macaque template.**

Note that only positive activations evoked by stimuli are shown. The borders of ROIs are encircled by white lines. The borders of V1, V2, and V4 are indicated by the black dashed lines.

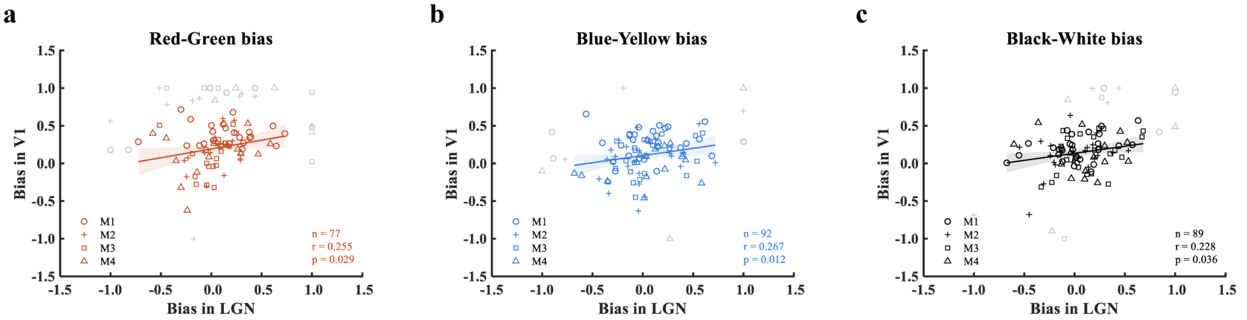

Supplementary Fig. 3 Correlation between LGN and V1 for bias indices after removal of extreme values.

a-c. Correlations between LGN and V1 for red–green bias (a), blue–yellow bias (b), and black–white bias (c) after removing data further away from the origin (two-tailed Spearman correlation, n of red-green bias = 77 runs, n of blue-yellow bias = 92 runs, n of black-white bias = 89 runs). Extreme bias indices (absolute value surpassed the threshold: 0.75) are marked with gray color. Each monkey is indicated by a different symbol. The shaded region represents 95% confidence intervals. Data are presented as mean +/- SE.

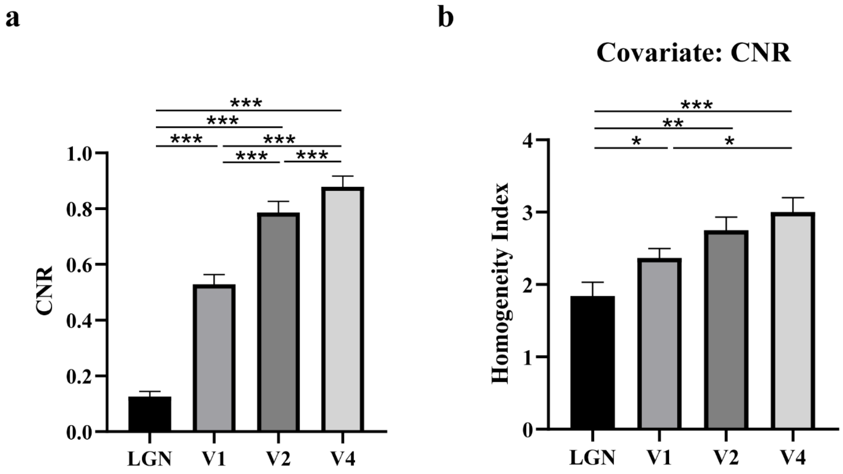

Supplementary Fig. 4 The contrast-to-noise ratio (CNR) of the LGN, V1, V2, and V4 and the change in the homogeneity index when CNR is treated as a covariate.

a. The contrast-to-noise ratio (CNR) in LGN, V1, V2, and V4. **b.** Differences in the homogeneity index among four regions after CNR was treated as a covariate. *$p < 0.05$, **$p < 0.01$, ***$p < 0.001$, post hoc comparisons of the generalized linear mixed model (GLMM) with Bonferroni corrections. Data are presented as mean +/- SE (n = 102 runs).

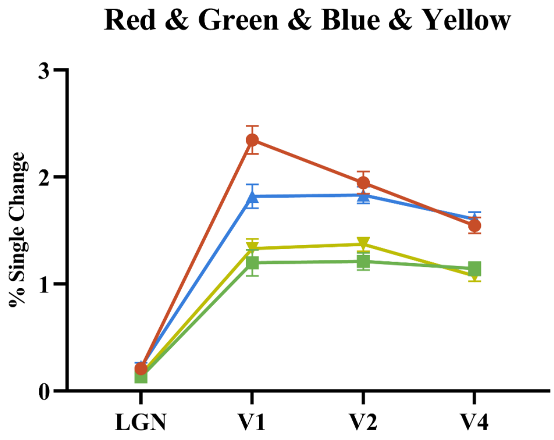

Supplementary Fig. 5 fMRI responses evoked by the six stimuli in the four ROIs.

Each stimulus is represented by its corresponding color. Data are presented as mean +/- SE (n = 102 runs).

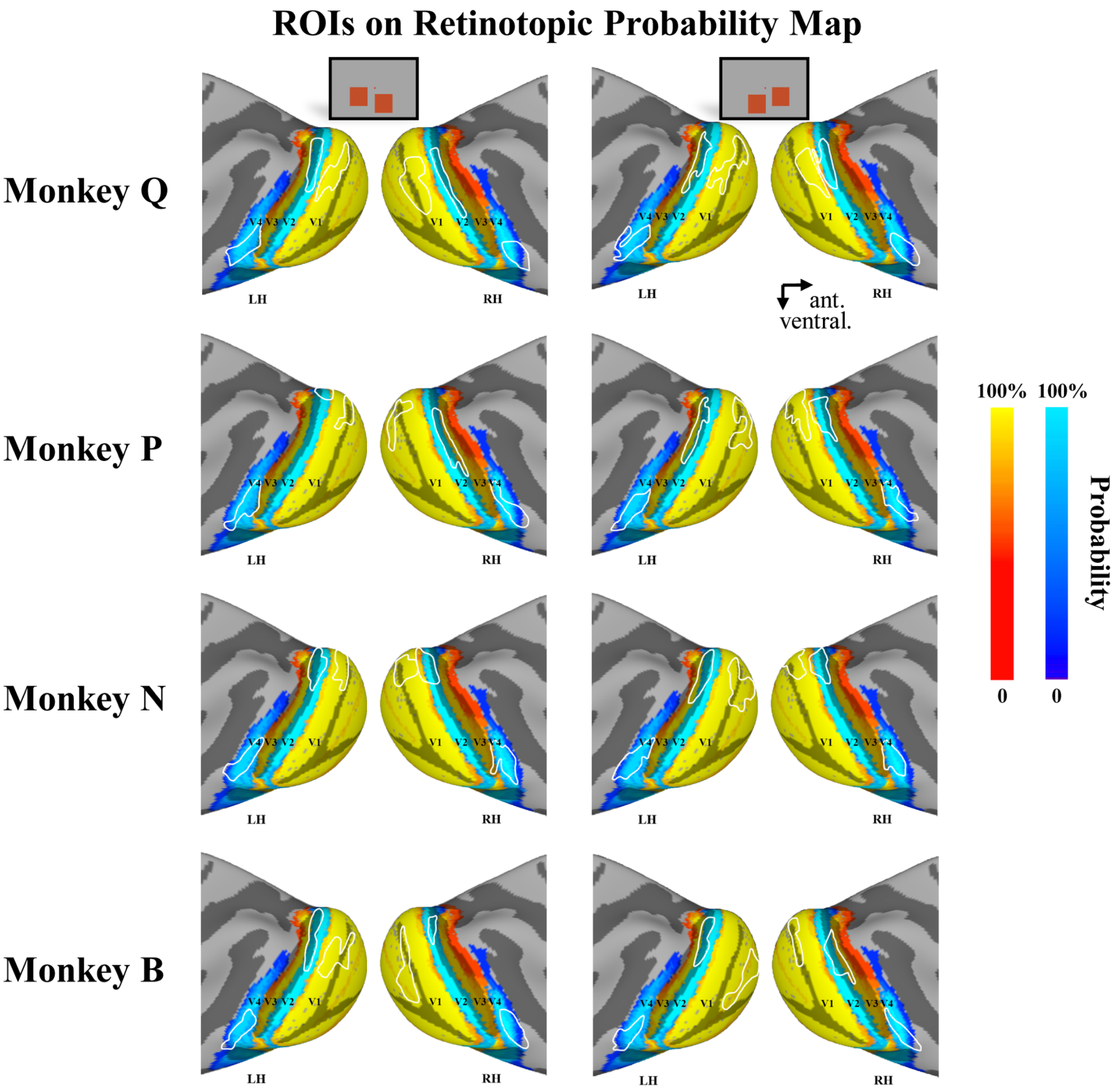

Supplementary Fig. 6. **The probabilistic map of V1, V2, V3, and V4 based on the dataset shared by Janssens et al. (2014) shown on lateral views of the inflated cortex of the macaque template.**

Note that voxels around the boundary are assigned to a visual region with the highest probability based on their probability in each region. The warm and cold colors represent V1/V3, and V2/V4, respectively, with the intensity of the color indicating the probability of the voxel representing the respective area. The borders of ROIs are encircled by white lines. The dataset shared by Janssens et al. (2014) was downloaded from https://gbiomed.kuleuven.be/english/research/50000666/50000669/50488669/neuroserv/publications/JNEUROSCI/index.html. The permission to use dataset from Janssens et al. 2014 (https://www.jneurosci.org/content/34/31/10156) has been granted by the corresponding author, Wim Vanduffel.

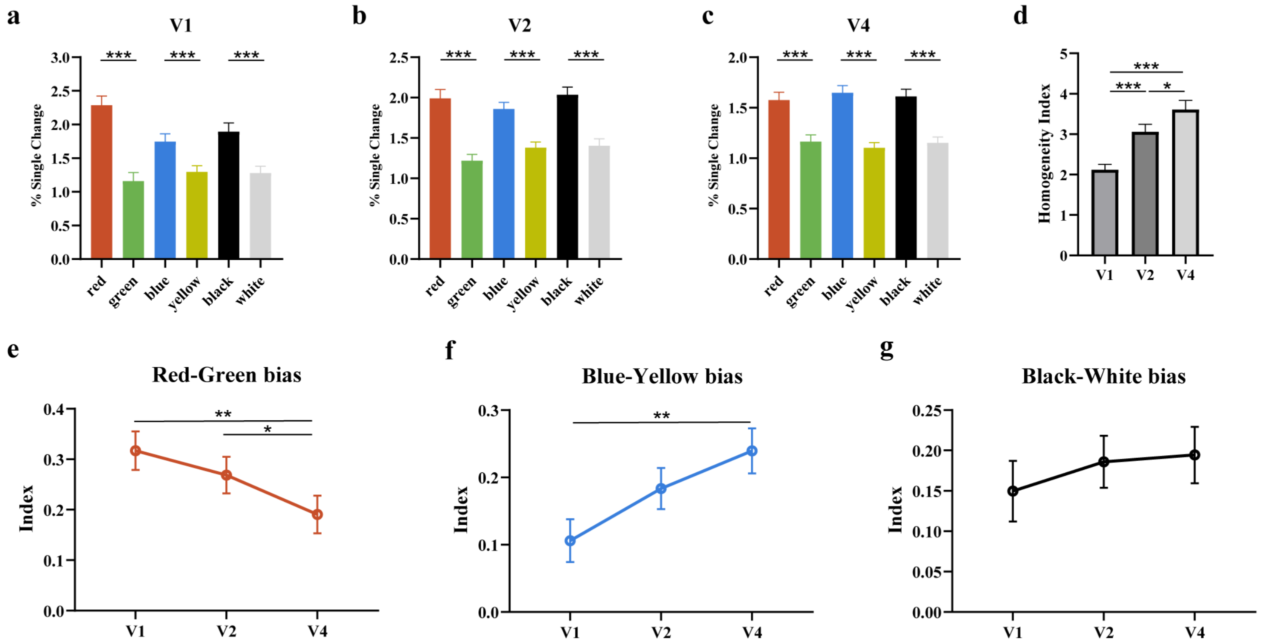

Supplementary Fig. 7. **End-spectral and polarity bias in V1, V2, and V4 based on voxels demonstrating at least 75% probability within the defined ROIs in the main text.**

a-c. The averaged fMRI responses to three color pairs across all four subjects in V1, V2, and V4, respectively. ***p < 0.001, GLMM analysis. **d.** The color homogeneity across V1, V2, and V4. *p<0.05, ***p < 0.001, post hoc comparisons of GLMM with Bonferroni corrections. **e-g.** The change trends of red–green bias, blue–yellow bias, and black–white bias from V1 to V4. *p < 0.05, **p < 0.01, post hoc comparisons of GLMM with Bonferroni corrections. Data are presented as mean +/- SE (n = 102 runs).

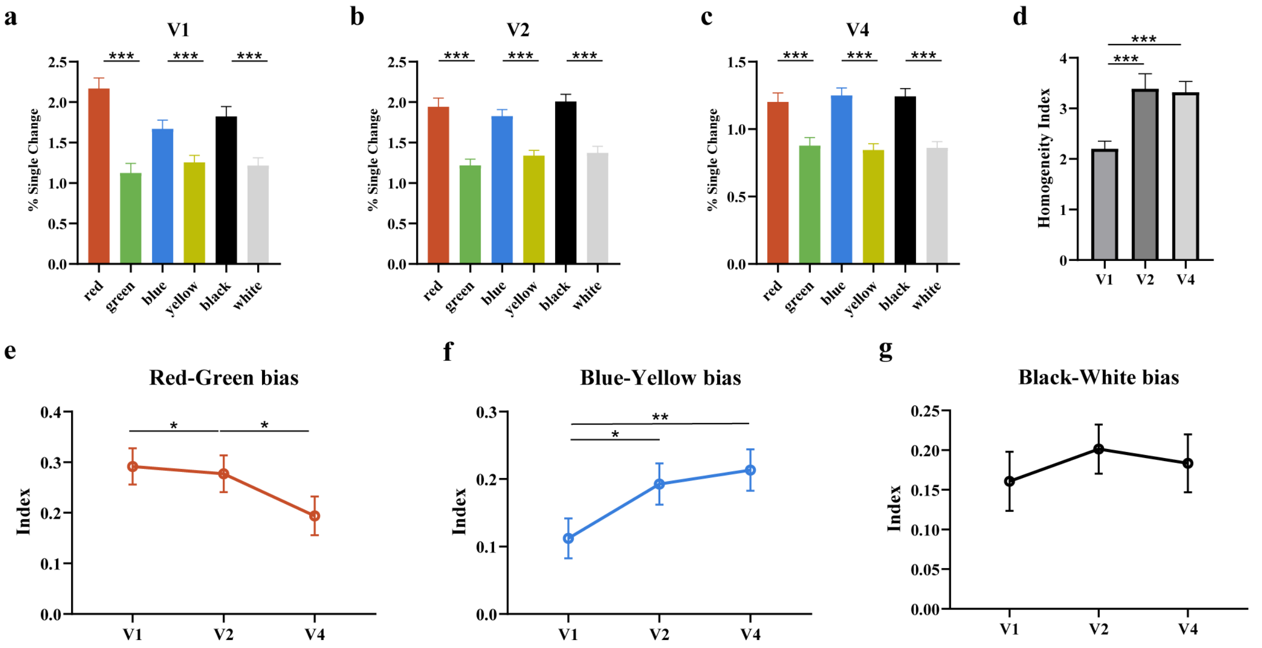

Supplementary Fig. 8. **End-spectral and polarity bias in V1, V2, and V4 based on the defined ROIs based on 75% probability V1/V2/V4 masks.**

a-c. The averaged fMRI responses to three color pairs across all four subjects in V1, V2, and V4, respectively. ***$p < 0.001$, GLMM analysis. **d.** The color homogeneity across V1, V2, and V4. ***$p < 0.001$, post hoc comparisons of GLMM with Bonferroni corrections. **e-g.** The change trends of red–green bias, blue–yellow bias, and black–white bias from V1 to V4. *$p < 0.05$, **$p < 0.01$, post hoc comparisons of GLMM with Bonferroni corrections. Data are presented as mean +/- SE (n = 102 runs).

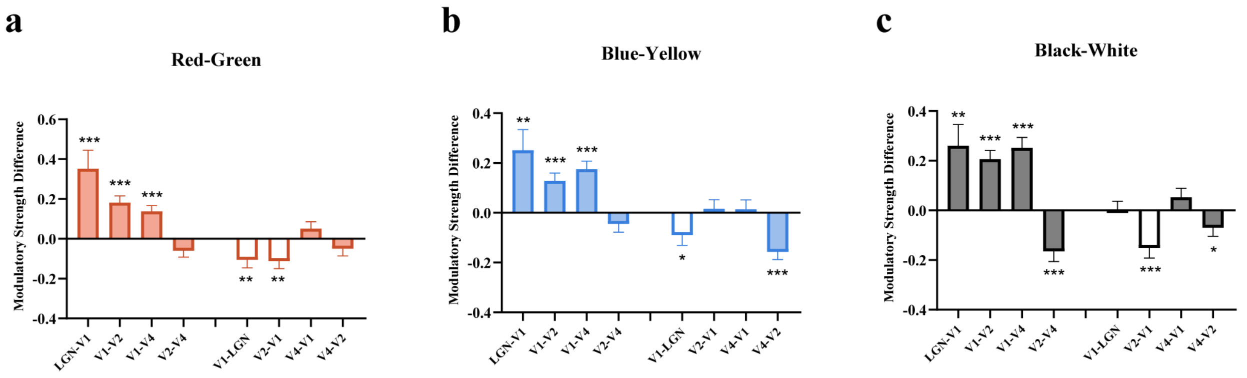

Supplementary Fig. 9 Differences between all feedforward and feedback modulatory connections in three pairs.

a-c. Differences between modulatory connections evoked by the two stimuli in red–green (a), blue–yellow (b), and black–white (c) pairs. **$p < 0.01$, ***$p < 0.001$ in comparison of GLMM analysis. Data are presented as mean +/- SE (n = 102 runs).

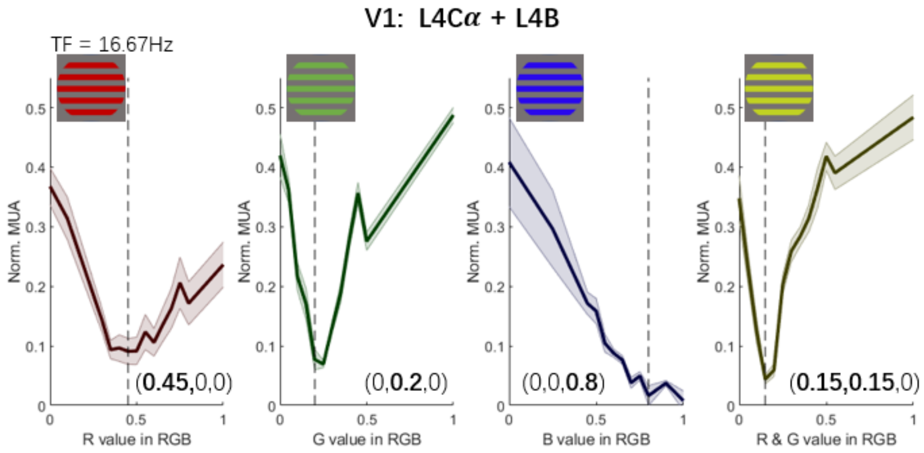

Supplementary Fig. 10 Response from V1 layers 4Cα and 4B in the luminance-matching experiment.

In the luminance-matching experiment, we used a fast square-wave drifting grating (SF = 2 Hz, temporal frequency = 16.67 Hz, preferred orientation) composed of background gray (10.6 cd/m2) and red/green/blue/yellow with multiple levels of luminance. Dashed vertical black lines indicate the RGB values (also labeled in the figure panels) in the x-axis corresponding to the minimal neural response. These RGB values are used in the control laminar electrophysiology experiment.

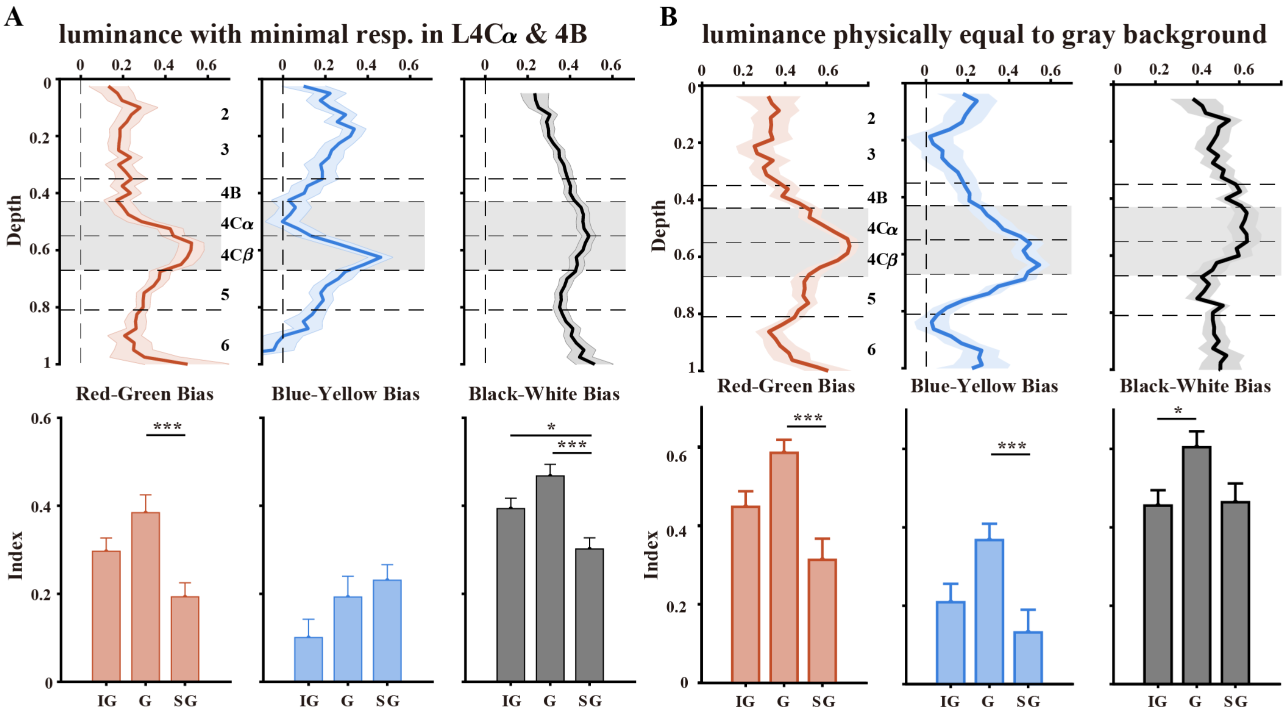

Supplementary Fig. 11 Laminar pattern of end-spectral bias and black dominance by using two sets of stimuli.

a. Laminar results of luminance matched by the M pathway (IG n = 87 channels, G n = 73 channels, SG n = 88 channels). **b.** Laminar results of physically equal luminance (same as Figure 5, IG n = 94 channels, G n = 89 channels, SG n = 83 channels). $^{*}p < 0.05$, $^{***}p < 0.001$ in Bonferroni-corrected post hoc comparisons following one-way ANOVA. Data are presented as mean +/- SE.

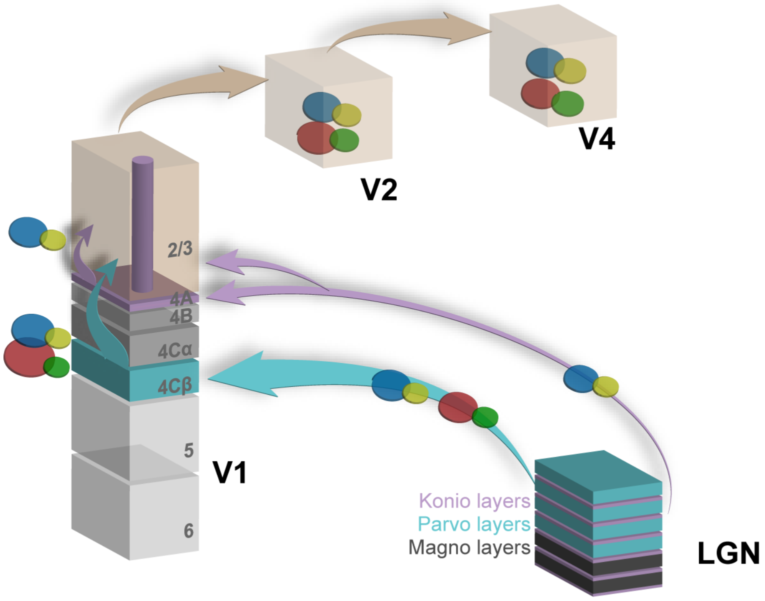

Supplementary Fig. 12 Neural origin and cortical modulation of end-spectral bias

In the schematic demonstration, the LGN of the primate provides unbalanced feedforward drives when primates (monkeys or human beings) are watching iso-luminance colors of red, green, blue, and yellow. The red bias and blue bias, conveyed by parvocellular pathway and koniocellular pathway, reached distinct layers in V1 and evoked similar end-spectral bias. The coding asymmetry of colors is further modulated through cortical processing both within columns and at multiple visual stages along the hierarchy.

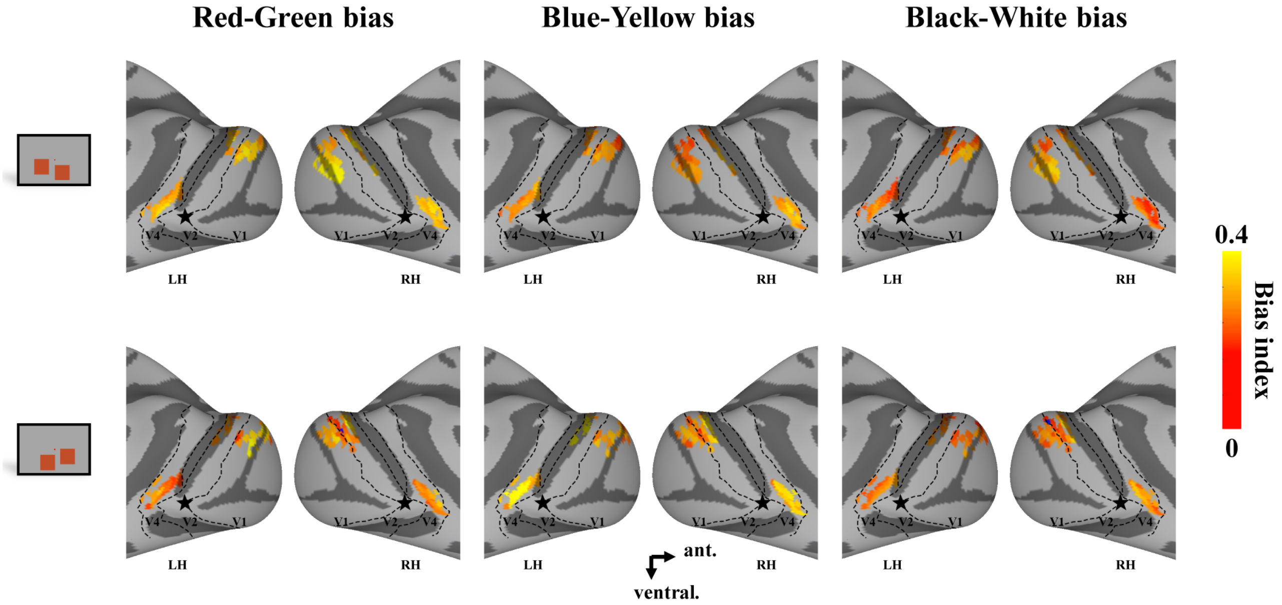

Supplementary Fig. 13 The distribution of bias indices in defined ROIs.

Three kinds of bias indices (from left to right, red–green bias, blue–yellow bias, black–white bias) for each voxel within each ROI of monkey Q are shown on lateral views of the inflated cortex of the macaque template. Black star marks the approximate location of the fovea based on the dataset shared by Janssens et al., (2014). The borders of V1, V2, and V4 are indicated by the black dashed lines. The dataset shared by Janssens et al. (2014) was downloaded from https://gbiomed.kuleuven.be/english/research/50000666/50000669/50488669/neuroserv/publications/JNEUROSCI/index.html. The permission to use dataset from Janssens et al. 2014 (https://www.jneurosci.org/content/34/31/10156) has been granted by the corresponding author, Wim Vanduffel.

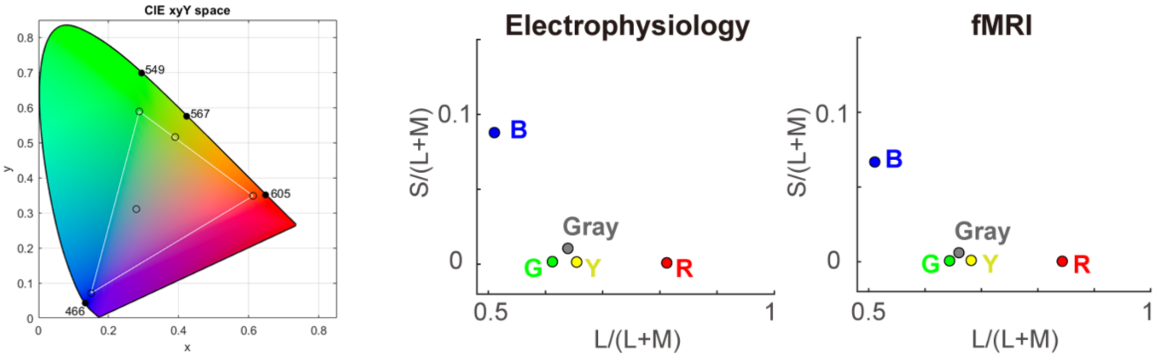

Supplementary Fig. 14 Stimuli of equal luminance in CIE 1931 space and in Macleod-Boynton color space

Left: Hollow circles represent equiluminant stimuli in CIE 1931 space used in the electrophysiological experiments. Solid circles represent the dominant wavelengths corresponding to each hue (blue: 466 nm, green: 549 nm, yellow: 567 nm, red: 605 nm). Right: equiluminant stimuli in Macleod-Boynton color space.

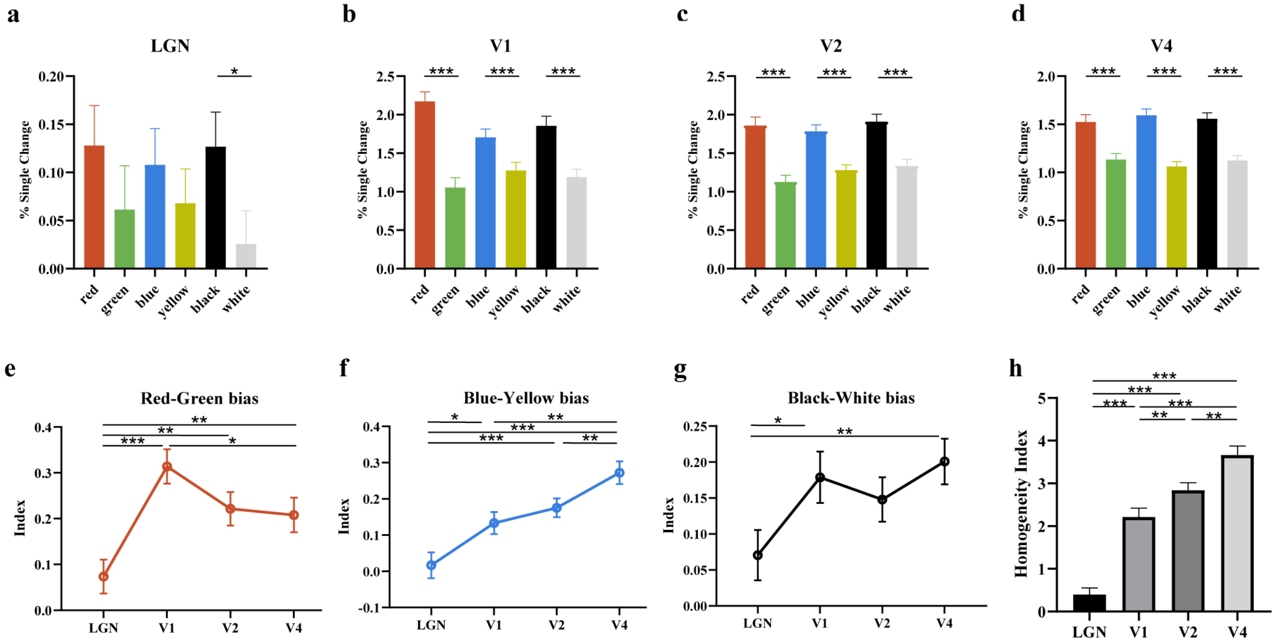

Supplementary Fig. 15 fMRI responses to each stimulus, bias indices, and homogeneity indices among the LGN, V1, V2, and V4 when defining ROIs using one-half of the data (odd or even runs) and testing on the other half.

a-d. Averaged fMRI responses to three color pairs across all four subjects in the LGN (a), V1 (b), V2 (c), and V4 (d). *$p < 0.05$, **$p < 0.01$, ***$p < 0.001$, GLMM analysis. **e-g.** The change trends of red–green bias (e), blue–yellow bias (f), and black–white bias (g) from LGN to V4. *$p < 0.05$, **$p < 0.01$, ***$p < 0.001$, post hoc comparisons of GLMM with Bonferroni corrections. **h.** Color homogeneity across visual areas. **$p < 0.01$, ***$p < 0.001$, post hoc comparisons of GLMM with Bonferroni corrections. Error bars indicate standard error. ROIs were defined using one-half of the data (odd or even runs), and responses were checked on the other half. Data are presented as mean +/- SE (n = 102 runs).

References

1 Bone, R. A. & Landrum, J. T. Heterochromatic flicker photometry. *Arch Biochem Biophys* **430**, 137-142, doi:10.1016/j.abb.2004.04.003 (2004).

2 Ives, F. E. A new color meter. *J Frankl Inst* **164**, 0047-0056, doi:Doi 10.1016/S0016-0032(07)90164-7 (1907).

3 Janssens, T., Zhu, Q., Popivanov, I. D. & Vanduffel, W. Probabilistic and single-subject retinotopic maps reveal the topographic organization of face patches in the macaque cortex. *J Neurosci* **34**, 10156-10167, doi:10.1523/JNEUROSCI.2914-13.2013 (2014).
