# Supplementary material for: The neural origin for asymmetric coding of surface color in the primate visual cortex
Source: Nat Commun. 2024 Jan 15;15:516. doi: 10.1038/s41467-024-44809-y (PMC10789876; doi:10.1038/s41467-024-44809-y)
Supplement: Supplementary file 3 — Reporting Summary [file 41467_2024_44809_MOESM3_ESM.pdf]

nature portfolio

Corresponding author(s): Ning Liu; Dajun Xing

Last updated by author(s): Nov 18, 2023

Reporting Summary

Nature Portfolio wishes to improve the reproducibility of the work that we publish. This form provides structure for consistency and transparency in reporting. For further information on Nature Portfolio policies, see our Editorial Policies and the Editorial Policy Checklist.

Statistics

For all statistical analyses, confirm that the following items are present in the figure legend, table legend, main text, or Methods section.

n/a	Confirmed	
☐	☒	The exact sample size (*n*) for each experimental group/condition, given as a discrete number and unit of measurement
☐	☒	A statement on whether measurements were taken from distinct samples or whether the same sample was measured repeatedly
☐	☒	The statistical test(s) used AND whether they are one- or two-sided<br>*Only common tests should be described solely by name; describe more complex techniques in the Methods section.*
☐	☒	A description of all covariates tested
☐	☒	A description of any assumptions or corrections, such as tests of normality and adjustment for multiple comparisons
☐	☒	A full description of the statistical parameters including central tendency (e.g. means) or other basic estimates (e.g. regression coefficient) AND variation (e.g. standard deviation) or associated estimates of uncertainty (e.g. confidence intervals)
☐	☒	For null hypothesis testing, the test statistic (e.g. *F*, *t*, *r*) with confidence intervals, effect sizes, degrees of freedom and *P* value noted<br>*Give P values as exact values whenever suitable.*
☒	☐	For Bayesian analysis, information on the choice of priors and Markov chain Monte Carlo settings
☒	☐	For hierarchical and complex designs, identification of the appropriate level for tests and full reporting of outcomes
☐	☒	Estimates of effect sizes (e.g. Cohen's *d*, Pearson's *r*), indicating how they were calculated

Our web collection on statistics for biologists contains articles on many of the points above.

Software and code

Policy information about availability of computer code

Data collection	Raw data were acquired by a 128-channel system (Blackrock Microsystems) and raw imaging data were collected in a 3T Siemens Prisma MRI scanner with a surface coil array (eight elements)
Data analysis	Functional data were preprocessed using Analysis of Functional NeuroImages software (AFNI), custom code was written using MATLAB (v.2018b), and IBM SPSS (v24) were used to conducted GLMM.

For manuscripts utilizing custom algorithms or software that are central to the research but not yet described in published literature, software must be made available to editors and reviewers. We strongly encourage code deposition in a community repository (e.g. GitHub). See the Nature Portfolio guidelines for submitting code & software for further information.

Data

Policy information about availability of data

All manuscripts must include a data availability statement. This statement should provide the following information, where applicable:

- Accession codes, unique identifiers, or web links for publicly available datasets
- A description of any restrictions on data availability
- For clinical datasets or third party data, please ensure that the statement adheres to our policy

The data that support the findings of this study are available from the corresponding author upon reasonable request.

Research involving human participants, their data, or biological material

Policy information about studies with human participants or human data. See also policy information about sex, gender (identity/presentation), and sexual orientation and race, ethnicity and racism.

Reporting on sex and gender	N/A
Reporting on race, ethnicity, or other socially relevant groupings	N/A
Population characteristics	N/A
Recruitment	N/A
Ethics oversight	N/A

Note that full information on the approval of the study protocol must also be provided in the manuscript.

Field-specific reporting

Please select the one below that is the best fit for your research. If you are not sure, read the appropriate sections before making your selection.

☒ Life sciences ☐ Behavioural & social sciences ☐ Ecological, evolutionary & environmental sciences

For a reference copy of the document with all sections, see nature.com/documents/nr-reporting-summary-flat.pdf

Life sciences study design

All studies must disclose on these points even when the disclosure is negative.

Sample size	No statistical methods were used to predetermine sample sizes but our sample sizes are chosen based on our previous publications (Yang et al., 2022; Wu et al., 2022). We did electrophysiological recording on four monkeys and imaging experiment on another four monkeys.
Data exclusions	In electrophysiological experiment, data from those runs in which fixation was maintained on less than 90% of the runs were excluded. In fMRI experiment, data were included from only those runs/trials in which fixation was maintained well [mean (SE): 88.08% (0.86%)].
Replication	We replicated our findings of end-spectral bias in all monkeys so we think the findings are reproducible. We have provided individual result of each monkey in the supplementary materials.
Randomization	No experimental groupings were used in this study.
Blinding	No experimental groupings were used in this study.

Reporting for specific materials, systems and methods

We require information from authors about some types of materials, experimental systems and methods used in many studies. Here, indicate whether each material, system or method listed is relevant to your study. If you are not sure if a list item applies to your research, read the appropriate section before selecting a response.

Materials & experimental systems

n/a	Involved in the study	
☒	☐	Antibodies
☒	☐	Eukaryotic cell lines
☒	☐	Palaeontology and archaeology
☐	☒	Animals and other organisms
☒	☐	Clinical data
☒	☐	Dual use research of concern
☒	☐	Plants

Methods

n/a	Involved in the study	
☒	☐	ChIP-seq
☒	☐	Flow cytometry
☐	☒	MRI-based neuroimaging

Animals and other research organisms

Policy information about studies involving animals; ARRIVE guidelines recommended for reporting animal research, and Sex and Gender in Research

Laboratory animals	Eight male adult rhesus monkeys (Macaca mulatta, 5-10 years, 4-11.5 Kg).
Wild animals	The study did not involve wild animals.
Reporting on sex	All eight monkeys are male.
Field-collected samples	The study did not involve samples collected from field.
Ethics oversight	All procedures were conducted in compliance with the National Institutes of Health Guide for the Care and Use of Laboratory Animals, and were approved by the Institutional Animal Care and Use Committee of Beijing Normal University and the Institutional Animal Care and Use Committee of Institute of Biophysics, CAS (IBP).

Note that full information on the approval of the study protocol must also be provided in the manuscript.

Plants

Seed stocks	N/A
Novel plant genotypes	N/A
Authentication	N/A

Magnetic resonance imaging

Experimental design

Design type	Event-related task design
Design specifications	There were 20-30 blocks per subject, 86 trials per block. Each stimulus presented for 2s per trial. Inter-trial interval was either 5.56s or a multiple of that duration when null trials occurred. Each block lasted 7 min and 58.16 s.
Behavioral performance measures	The monkeys were required to maintain fixation on the white fixation cross superimposed on the stimuli to receive a liquid reward. Data were included from only those runs/trials in which fixation was maintained well [mean (SE): 88.08% (0.86%)]

Acquisition

Imaging type(s)	Functional MRI
Field strength	3 T
Sequence & imaging parameters	Imaging parameters were as follows: voxel size: 1.5 mm isotropic, field of view: 129 × 129 mm; matrix size: 86 × 86; echo time (TE): 17 ms; repetition time (TR): 1.39 s; flip angle: 90°. A low-resolution T2 anatomical scan was also acquired in each session to serve as an anatomical reference (0.625 mm×0.625 mm×1.5 mm; TE: 101 ms; TR: 11.200 s; flip angle: 126°). To facilitate alignment to the template, we also acquired high-resolution T1-weighted whole-brain anatomical scans in separate sessions. Imaging parameters were as follows: voxel size: 0.5 mm isotropic; TE: 2.84 ms; TR: 2.2 s; flip angle: 8°.
Area of acquisition	Twenty-seven 1.5-mm coronal slices (no gap) were acquired using single-shot interleaved gradient-recalled echo planar imaging.

Diffusion MRI ☐ Used ☒ Not used

Preprocessing

Preprocessing software	Functional data were preprocessed using Analysis of Functional NeuroImages software (AFNI). The data were smoothed with a 2-mm full-width half-maximum Gaussian kernel

Normalization	Images were realigned to the base volume of one initial localizer session. Linear transformation were conducted.
Normalization template	We aligned statistical map images onto images of the symmetric NIMH Macaque Template (NMT) v2, and also projected results onto a rendered and inflated version of the NMT v2 cortical surface.
Noise and artifact removal	For each voxel, we performed a single univariate linear model fit to estimate the response amplitude for each condition. The model included a hemodynamic response predictor for each category and regressors of no interest (baseline, movement parameters, and signal drifts).
Volume censoring	Volumes where more than 0.5% of the brain voxels were computed as outliers were censored. No volume was censored out in the remained runs.

Statistical modeling & inference

Model type and settings	For each voxel, we performed a single univariate linear model fit to estimate the response amplitude for each condition.
Effect(s) tested	Cluster-wise percent signal change for different stimuli.

Specify type of analysis: ☐ Whole brain ☒ ROI-based ☐ Both

Anatomical location(s): LGN, V1, V2, V4.

Statistic type for inference<br>(See Eklund et al. 2016)	We perform Generalized Linear Mixed Models (GLMMs) to compare responses to different stimuli or ROIs, with Monkey, Session, and Run as random factors.
Correction	When comparing responses of paired stimuli, no post hoc comparison is required. We used Boferroni correction when the number of conditions are more than two.

Models & analysis

n/a	Involved in the study
☐	☒ Functional and/or effective connectivity
☒	☐ Graph analysis
☐	☒ Multivariate modeling or predictive analysis

Functional and/or effective connectivity	Pearson correlation
Multivariate modeling and predictive analysis	Dynamic causal modeling
